# Social Franchising: A Blockbuster to Address Unmet Need for Family Planning and to Advance Toward the FP2020 Goal

**DOI:** 10.9745/GHSP-D-15-00155

**Published:** 2015-06-08

## Abstract

Social franchising has scaled-up provision of voluntary family planning, especially long-acting reversible contraceptives, across Africa and Asia at a rapid and remarkable pace. The approach should be pursued vigorously, especially in countries with a significant private-sector presence, to advance the FP2020 goal of providing access to modern contraception to 120 million additional clients by 2020.

See related articles by Munroe and by Thurston.

In this issue of GHSP, we showcase 2 landmark articles on family planning social franchising. Social franchising differs from social marketing by focusing on providing a *service* (typically a clinical service), such as inserting and removing contraceptive implants, along with providing the accompanying needed products. It taps the vast resource of largely small-scale private health outlets in developing countries. While the approach has been around for some time, Population Services International (PSI) and Marie Stopes International (MSI) have “honed” the model to a highly successful, keen intervention.

## HOW DO THEY DO IT?

Among the key ingredients described by Thurston and colleagues^1^ are:

1. **Amenable settings** with an adequate private sector already serving low-income clients and a favorable government policy environment, including a positive climate for task sharing
2. **Motivated providers**, often female, supportive of reproductive health services and well-located in low-income areas with little overlap with other reproductive health providers
3. Key support including: competency-based clinical **training**, minimum **standards**, **supplies**, **supervision**, and a variety of **quality assurance** mechanisms
4. A franchise **“brand”**
5. Support for providers to **build their business** though business management training and by selectively providing affordable capital
6. **Demand creation** and selective use of financial mechanisms such as vouchers to enhance access for those least able to pay
7. **Advocacy** with governments on issues such as task shifting and linkage with health insurance schemes
8. Good **service products** including long-acting reversible contraceptives (**LARCs**)—implants and IUDs—which are both popular among clients and conducive to the skill level of many private providers

## MAJOR PROGRESS TOWARD THE FP2020 GOAL

What has been accomplished? An amazing amount. Munroe and colleagues^2^ report detail on MSI’s efforts only. But Thurston et al. report on couple-years of protection (CYPs) for both PSI and MSI combined. The CYPs they provided through social franchising grew rapidly—remarkably, by roughly 25% in just 1 year, from 8.6 million to 10.8 million between 2013 and 2014. If the annual growth going forward were an admittedly optimistic 25%, then by 2020 CYPs provided in that year alone would be over 40 million! Clearly this effort is destined to be pivotal for serving great numbers of women and for helping to reach the challenging Family Planning 2020 (FP2020) goal of providing access to modern contraception to 120 million additional clients by 2020.^3^

## BUT WAIT, THERE'S MORE

Munroe et al. describe many other positive accomplishments of MSI’s efforts:

- A high proportion of clients were **young** women—26.1% aged 15–24
- A high proportion were **low-income** women—57.4% living on under US$2.50 per day
- A high proportion were **“new” clients**—40.7% (in the sense that they had not used a modern method in the prior 3 months), thus indicating MSI is reaching clients otherwise often not reached and is squarely addressing unmet need
- A very high proportion of clients selected **LARCs**—68% by 2014
- **Quality** promotion, including improving quality audit scores over time
- High reported client **satisfaction**, with a mean score of 4.51 out of a possible 5 (although we know social desirability bias may likely yield high satisfaction scores in any case)

68% of MSI social franchising clients chose LARCs.

## WHERE NEXT?

First, keep going with a winner and continue to scale-up. Achievement of FP2020 goals will rise or fall depending on success in key countries such as Nigeria, the Democratic Republic of the Congo, Ethiopia, and Pakistan. *Nigeria, in particular, should be a major priority.* This largest country in Africa has thus far shown little progress in family planning, but it appears especially conducive to wide expansion of social franchising in view of its very high reliance on private-sector providers for health services. Other countries with less private-sector presence will need a different strategic program mix, notably including emphasis on mobile outreach, which has also demonstrated remarkable success.^4^

Nigeria, with its high reliance on the private sector, may be especially conducive to wide expansion of social franchising.

### Challenges

Continued growth and scale-up always present important challenges. Over time, both PSI and MSI have made many improvements and adjustments. Incremental improvements in efficiency, quality, and quantity should continue to accrue through experience and additional innovation. Maintaining **quality**, including promoting wide choice of methods, is always challenging with rapid scale-up, especially realizing private providers remain largely free agents. Franchisors must develop additional quality assurance mechanisms that are replicable and cost-effective. Thurston and colleagues describe strategies that MSI and PSI are testing, including new use of technology. Additional challenges are continued progress in reaching **youth and low-income** women.

Promoting quality, including ensuring a wide choice of methods, is important.

And **sustainability** over the longer term must always be on the agenda. The effort so far has required substantial donor support. But family planning is such a good long-term investment for individuals and society that a large degree of donor subsidy is fully justified for a long period of time in many countries, in return for such impressive results. Nevertheless, one attraction of a commercial-sector model is the possibility of recouping costs and eventually even long-term cost self-sufficiency. After all, the history of family planning in Latin America saw an evolution from subsidized social marketing though such organizations as Profamilia in Colombia, to out-and-out true commercial marketing of contraceptives, notably oral contraceptives, that continues to this day. As Thurston et al. point out, additional ways of addressing sustainability are broadening services beyond family planning (although that would need to be done selectively and with care) and coverage under health insurance schemes, whether considered under the label of universal health coverage or otherwise.

Beyond MSI and PSI, many other organizations are taking up social franchising for family planning and for other worthy health objectives.^5^ The global health community should pursue this programmatic “best bet” vigorously. *–Global Health: Science and Practice*

## References

[1] Thurston S Chakraborty N Hayes B Mackay A Moon P Establishing and scaling-up clinical social franchise networks: lessons learned from Marie Stopes International and Population Services International. Glob Health Sci Pract. 2015;3(2). 10.9745/GHSP-D-15-00057 PMC4476858 26085017

[2] Munroe E Hayes B Taft J Private-sector social franchising to accelerate family planning access, choice, and quality: results from Marie Stopes International. Glob Health Sci Pract. 2015;3(2). 10.9745/GHSP-D-15-00056 PMC4476859 26085018

[3] Brown W Druce N Bunting J Radloff S Koroma D Gupta S Developing the “120 by 20” goal for the Global FP2020 initiative. Stud Fam Plann. 2014;45(1): 73–84. 10.1111/j.1728-4465.2014.00377.x. 24615576

[4] Duvall S Thurston S Weinberger M Nuccio O Fuchs-Montgomery N. Scaling up delivery of contraceptive implants in sub-Saharan Africa: operational experiences of Marie Stopes International. Glob Health Sci Pract. 2014;2(1): 72–92. 10.9745/GHSP-D-13-00116. 25276564 PMC4168608

[5] Viswanathan R Schatzkin E Sprockett A Clinical social franchising compendium: an annual survey of programs: findings from 2013. San Francisco (CA): University of California, San Francisco, Global Health Sciences, The Global Health Group; 2014 Available from: http://sf4health.org/sites/sf4health.org/files/wysiwyg/Social-Franchising-Compendium-2014_0.pdf

